# *Trichomonas vaginalis* Macrophage Migration Inhibitory Factor Mediates Parasite Survival during Nutrient Stress

**DOI:** 10.1128/mBio.00910-18

**Published:** 2018-06-26

**Authors:** Yi-Pei Chen, Olivia Twu, Patricia J. Johnson

**Affiliations:** a Molecular Biology Institute, University of California, Los Angeles, Los Angeles, California, USA; b Department of Microbiology, Immunology & Molecular Genetics, University of California, Los Angeles, Los Angeles, California, USA; Harvard T. H. Chan School of Public Health

**Keywords:** *Trichomonas vaginalis*, apoptosis, macrophage migration inhibitory factor, nutrient starvation

## Abstract

Trichomonas vaginalis is responsible for the most prevalent non-viral sexually transmitted disease worldwide, and yet the mechanisms used by this parasite to establish and maintain infection are poorly understood. We previously identified a T. vaginalis homologue (TvMIF) of a human cytokine, human macrophage migration inhibitory factor (huMIF). TvMIF mimics huMIF’s role in increasing cell growth and inhibiting apoptosis in human host cells. To interrogate a role of TvMIF in parasite survival during infection, we asked whether overexpression of TvMIF (TvMIF-OE) confers an advantage to the parasite under nutrient stress conditions by comparing the survival of TvMIF-OE parasites to that of empty vector (EV) parasites. We found that under conditions of serum starvation, overexpression of TvMIF resulted in increased parasite survival. Serum-starved parasites secrete 2.5-fold more intrinsic TvMIF than unstarved parasites, stimulating autocrine and paracrine signaling. Similarly, we observed that addition of recombinant TvMIF increased the survival of the parasites in the absence of serum. Recombinant huMIF likewise increased the parasite survival in the absence of serum, indicating that the parasite may use this host survival factor to resist its own death. Moreover, TvMIF-OE parasites were found to undergo significantly less apoptosis and reactive oxygen species (ROS) generation under conditions of serum starvation, consistent with increased survival being the result of blocking ROS-induced apoptosis. These studies demonstrated that a parasitic MIF enhances survival under adverse conditions and defined TvMIF and huMIF as conserved survival factors that exhibit cross talk in host-pathogen interactions.

## IMPORTANCE

Macrophage migration inhibitory factor (MIF) is a conserved protein found in most eukaryotes which has been well characterized in mammals but poorly studied in other eukaryotes. The limited analyses of MIF proteins found in unicellular eukaryotes have focused exclusively on the effect of parasitic MIF on the mammalian host. This was the first study to assess the function of a parasite MIF in parasite biology. We demonstrate that the Trichomonas vaginalis MIF functions to suppress cell death induced by apoptosis, thereby enhancing parasite survival under adverse conditions. Our research reveals a conserved survival mechanism, shared by a parasite and its host, and indicates a role for a conserved protein in mediating cross talk in host-pathogen interactions.

## INTRODUCTION

Trichomonas vaginalis, an extracellular unicellular parasite, causes the most common non-viral sexually transmitted infection in the world (1) but has been long neglected. Thus, the mechanisms that drive parasite pathogenesis and the disease epidemiology are poorly understood (2). However, this is changing as more molecular and genetic tools for analyses are developed (3, 4). The majority of T. vaginalis infections are asymptomatic. When they are symptomatic, the manifestations of infection vary greatly and may include inflammation of the urogenital tract, preterm delivery, and increased chances of HIV co-infection (5, 6). The factors that determine how the parasite maintains the infection in the vaginal microenvironment, where the nutrients, hormones, and pH are constantly changing, remain largely unknown (7–9).

Macrophage migration inhibitory factor (MIF) is a highly conserved eukaryotic protein found across unicellular protists, plants, arthropods, and mammals (10–13). Human MIF (huMIF) has been widely studied and is known to play essential roles in cell growth, survival and in cancer growth in humans (14–16). In our previous study, we reported that T. vaginalis shares a homologous protein (TvMIF) with the human host and that TvMIF can activate the same survival pathways as huMIF in human cells (17). MIF homologues found in other eukaryotic parasites are known to modulate the host immune system and to activate huMIF pathways (10, 18–21). Although the effects of parasite-derived MIFs from parasites on host cells have been studied, the role of MIF in parasites and non-mammalian systems is poorly understood.

T. vaginalis and other parasitic protists share certain apoptotic phenotypes with mammalian systems such as DNA fragmentation and phosphatidylserine exposure (22–25). However, no factor that either stimulates or suppresses apoptosis in these divergent, unicellular eukaryotes has yet been identified. In this report, we describe the anti-apoptotic effect caused by TvMIF, a conserved eukaryotic protein, and reveal similarities between this protein and its mammalian homologue. We also provide evidence that T. vaginalis is able to exploit huMIF to enhance its survival during nutrient starvation. These studies uncovered a highly conserved eukaryotic protein used by a parasite and its host to enhance survival.

## RESULTS

### T. vaginalis MIF (TvMIF) enhances survival of the parasites under conditions of nutrient stress.

We have previously shown that the homologue of huMIF in the parasite T. vaginalis (TvMIF) can induce the growth and activation of anti-apoptotic pathways in human host cells (17). To investigate whether TvMIF can perform a similar function and hence enhance the survival of the parasite under adverse conditions, we have studied the role of TvMIF in parasite survival under conditions of nutrient and density stress. T. vaginalis is typically grown in Diamond’s media supplemented with serum (26). The parasite will not grow in the absence of serum as it provides lipids, precursors of nucleotides, and amino acids required for parasite survival (26, 27). Parasites also cease to swim and die within hours after reaching a density of 5 × 10^6^ cells/ml in Diamond’s media supplemented with serum (27). As a first step toward determining whether TvMIF plays a role in parasite survival, we compared the survival rates of parasites transfected with (28) and overexpressing TvMIF (TvMIF-OE) with those transfected with an empty vector (EV). Immunoblotting confirmed that expression of TvMIF in TvMIF-OE parasites is approximately 17-fold greater than in EV control parasites (see Fig. S1 in the supplemental material).

10.1128/mBio.00910-18.1 FIG S1  Quantification of TvMIF levels in EV and TvMIF-OE parasites assessed by immunoblotting. (A) "Exo" and "Endo" indicate the exogenous TvMIF and endogenous TvMIF, respectively. GAPDH was used as a loading control. (B) TvMIF protein quantity is determined by normalizing to GAPDH and setting the EV TvMIF protein level as 1. The endogenous TvMIF level in the TvMIF-OE is ~3.93-fold and the exogenous TvMIF level is ~16.7-fold relative to the EV level. **, *P* value ≤ 0.01; ***, *P* value ≤ 0.001. All data represent results from 3 independent experiments, each done in triplicate. Download FIG S1, TIF file, 2.3 MB. Copyright © 2018 Chen et al. 2018 Chen et al. This content is distributed under the terms of the Creative Commons Attribution 4.0 International license.

**FIG 1:**
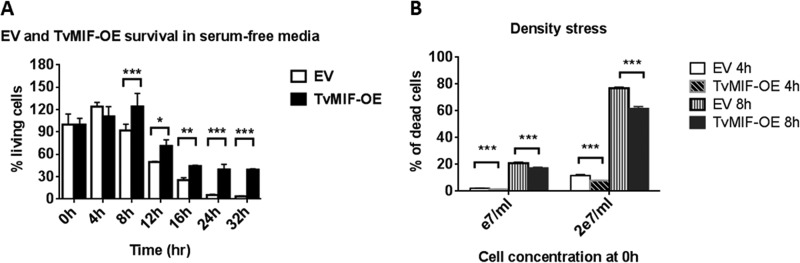
TvMIF increases parasite survival under conditions of nutrient stress. (A) Overexpression of TvMIF in the parasite enhances the survival of the parasite under conditions of serum starvation significantly after 8 h. All time points are normalized to time point 0 h for each parasite. Data are means ± standard errors of results from triplicates, and data from 1 of 3 independent experiments are shown. (B) Overexpression of TvMIF enhances parasite resistance to density stress. The cultures started at a 1 × e7/ml or 2 × e7/ml concentration and were incubated for 4 and 8 h. Data shown are means ± standard errors of results from triplicates, and data representative of 1 of 3 independent experiments are shown. *, *P* value ≤ 0.05; **, *P* value ≤ 0.01; ***, *P* value ≤ 0.001.

Fluorescence-activated cell sorter (FACS) analyses of living cells assessed using Zombie Red viability dye to compare TvMIF-OE and EV parasites were conducted using parasites grown in serum-free media or at a high density. We found that TvMIF-OE parasites survived significantly better than EV parasites in serum-free media after 8 h of incubation and that this survival phenotype became more pronounced after 24 to 32 h of starvation (Fig. 1A; see also Fig. S2). Comparison of the survival rates of TvMIF-OE and EV parasites at densities as high as 10^7^ cells/ml or 2 × 10^7^ cells/ml also revealed the death of a significantly higher proportion of EV parasites than of TvMIF-OE parasites at 4 and 8 h after they were subjected to density stress (Fig. 1B). When grown in regular media supplemented with serum, EV and TvMIF-OE had similar growth rates (Fig. S3), demonstrating that TvMIF promotes parasite survival under adverse conditions.

10.1128/mBio.00910-18.2 FIG S2  Gating strategy for survival assay. (A) Heat-inactivated control results showed that the dead cells were smaller (low forward scatter [FSC]) and had higher granularity (high side scatter [SSC]). By gating the dead population, we confirmed that Zombie Red viability dye stained positively on dead cells (PE-Texas Red positive). (B) Gating strategy for cells undergoing serum starvation or density stress. Gating of live population and counting beads showed that the live cells were stained as Zombie Red negative (PE-Texas Red negative). The counting beads were further gated using Alexa Fluor 405 positivity to exclude the dead cells in the gate. By normalizing the numbers of totally live cells to bead numbers, we could determine the percentage of the living population in each sample. Download FIG S2, TIF file, 2.2 MB. Copyright © 2018 Chen et al. 2018 Chen et al. This content is distributed under the terms of the Creative Commons Attribution 4.0 International license.

10.1128/mBio.00910-18.3 FIG S3  Growth comparison between EV and TvMIF-OE parasites in complete Diamond’s media containing serum. EV and TvMIF-OE parasites (4 × 10^4^ cells/ml) were cultured in complete Diamond’s media. At 12 h and 24 h, cells were counted by FACS analysis, and there was no significant growth difference at either time point. Error bars represent standard errors. Ns, non-significant. Download FIG S3, TIF file, 1.8 MB. Copyright © 2018 Chen et al. 2018 Chen et al. This content is distributed under the terms of the Creative Commons Attribution 4.0 International license.

### TvMIF is secreted as both a free soluble protein and an exosomal protein.

**FIG 2:**
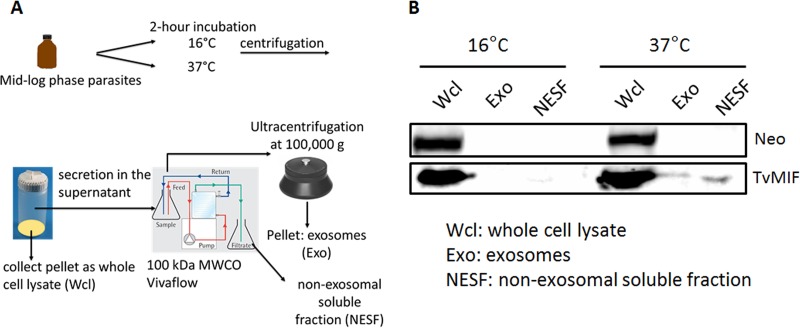
TvMIF is present in the exosome and the non-exosomal secreted fractions. (A) A secretion assay used to determine proportions of TvMIF in the exosome (Exo) and non-exosomal soluble fraction (NESF) is illustrated. Parasites were incubated at either 16°C or 37°C. Incubation at 16°C is a negative control for secretion. After the incubation, the cells were pelleted and lysed to become whole-cell lysates (Wcl) and the secreted supernatant fraction was passed through a Vivaflow crossflow cassette to separate Exo from NESF. Then, the Exo was collected by ultracentrifugation. (B) At 16°C, no detectable TvMIF was present in Exo and NESF. At 37°C, 1.4-fold more TvMIF was detected in the NESF than in the Exo fraction using immunoblotting and a TvMIF antibody. Neomycin phosphotransferase (Neo) was used to detect cellular lysis.

We have previously demonstrated that T. vaginalis secretes small vesicles called exosomes (29, 30) and that TvMIF is found in the exosomal proteome (31). As the survival pathways activated by huMIF require a secreted soluble form of the protein (16, 32), we examined both the exosomal fractions (Exo) and the non-exosomal soluble fractions (NESF) secreted by the parasite for TvMIF using a Vivaflow crossflow cassette and an ultracentrifugation-based method (Fig. 2A). Parasites grown overnight were collected and then incubated in phosphate-buffered saline (PBS)–5% sucrose at either 16°C (negative control for secretion) or 37°C. After 2 h of incubation, the cells were pelleted and lysed to make whole-cell lysates (Wcl). The supernatant was passed through a Vivaflow crossflow cassette using a 100-kDa molecular weight cutoff (MWCO) to separate the heavier exosomes (Exo > 100 kDa) from the lighter non-exosomal secreted fraction (NESF < 100 kDa). The level of NESF TvMIF was found to be 1.4-fold higher than that of Exo TvMIF by immunoblotting using an anti-TvMIF antibody (4) (Fig. 2B). As the Exo fraction had much less protein than the Wcl fraction and NESF from equal numbers of cells, equal (20 µg) amounts of protein were loaded in each fraction. At 37°C, 20 µg of protein is equal to 2.87% of the total NESF and 17.2% of the Exo fraction. As a result, we can reason that approximately 8.4-fold-more TvMIF is secreted in the NESF than in the Exo (17.2% divided by 2.87% and then multiplied by 1.4-fold). These data show that the majority of total secreted TvMIF is present as a soluble protein.

### Both intracellular TvMIF and secreted TvMIF are induced during serum starvation.

**FIG 3:**
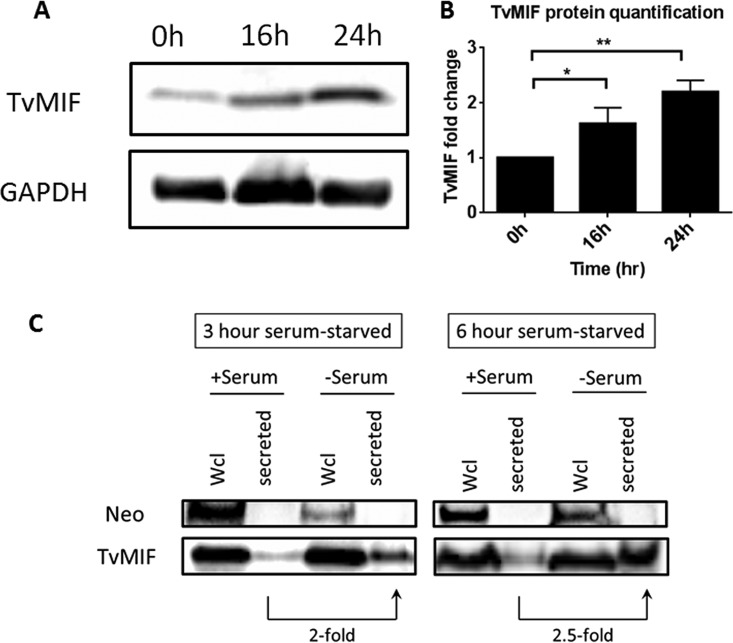
Endogenous TvMIF protein is induced under conditions of serum starvation. (A) Anti-TvMIF immunoblot showing that the TvMIF level was induced during serum starvation. Detection of GAPDH served as the loading control. One representative immunoblot of three independent experiments is shown. (B) Quantification of TvMIF was determined by normalizing the TvMIF signal to GAPDH for each sample; the data were compared to the 0-h time point value (set at 1) in each experiment, so no error bar was made for 0 h. Data are means ± standard errors. (C) TvMIF secretion was induced in the absence of serum. +Serum, cells grown in complete media; −Serum, cells grown without serum. Secreted TvMIF was induced ~2-fold at 3 h and ~2.5-fold at 6 h after serum starvation comparing −serum and +serum signals. Neo was used as a negative control for cell lysis.

As shown in Fig. 1, overexpression of TvMIF increased the survival of the parasites during nutrient stress. Thus, we asked if induction of TvMIF expression and its secretion play a role in the enhanced survival. First, to test if intracellular TvMIF expression is induced under conditions of serum starvation, we collected the whole-cell lysates (Wcl) from cells starved for 16 or 24 h and TvMIF levels were quantified by immunoblotting. Intracellular TvMIF was found to be induced 1.6-fold at 16 h and 2.2-fold at 24 h after starvation (Fig. 3A and B). For determining TvMIF secretion levels under conditions of serum starvation, parasites grown overnight in complete Diamond’s media were collected, resuspended, and incubated in serum-free media for 3 h or 6 h at 37°C. Cells were then spun, resuspended in PBS–5% sucrose, and incubated for an additional 2 h at 37°C. Cells were then pelleted and lysed to make Wcl, and the supernatant was collected to assess secretion. Immunoblot analyses showed that the secreted TvMIF were induced ~2-fold and 2.5-fold higher from cells incubated in serum-free media for 3 and 6 h, respectively, than from parasites grown in serum-containing media (Fig. 3C). Neomycin phosphotransferase (Neo), a non-secreted protein, was used as a control to monitor cell lysis. The presence of Neo signal only in the Wcl confirms that the TvMIF signal detected was the result of secretion and not cell lysis.

### Addition of recombinant MIFs increases survival of the parasite during serum starvation.

**FIG 4:**
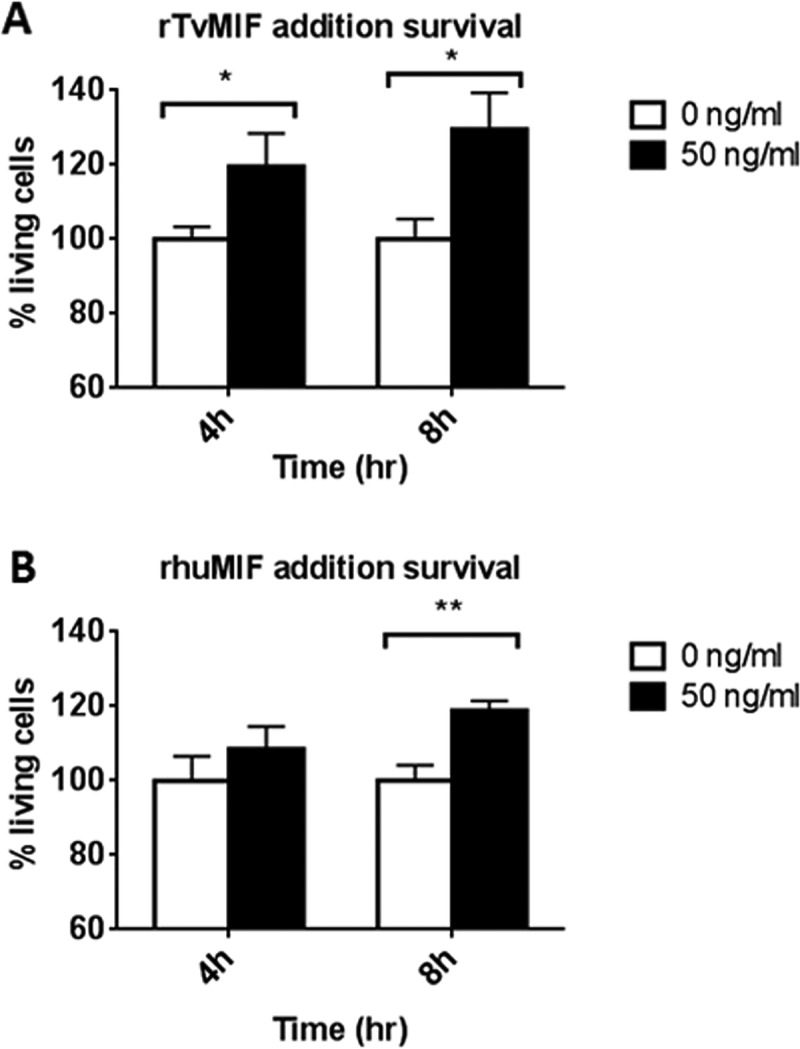
Both recombinant TvMIF and recombinant huMIF increase the survival of the parasite under conditions of serum starvation. (A) rTvMIF (50 ng/ml) or PBS (indicated as 0 ng/ml) was added to parasites grown without serum at time point 0 h. rTvMIF at 50 ng/ml increased the parasite survival at both 4 h and 8 h after serum starvation. (B) rhuMIF (50 ng/ml) or PBS (indicated as 0 ng/ml) was added to parasites grown without serum at time point 0 h. rhuMIF at 50 ng/ml increased the parasite survival at 8 h after starvation. Results of 3 independent experiments, each done in quadruplet, are represented by the data shown here. Error bars represent means ± standard errors. *, *P* value ≤ 0.05; **, *P* value ≤ 0.01.

As survival and anti-apoptotic pathways are activated by huMIF in an autocrine manner (15, 33, 34) and TvMIF secretion is induced during serum starvation (Fig. 3), we generated recombinant MIF and added it to parasites to directly test whether soluble TvMIF plays a role in enhancing parasite survival during serum starvation. As shown in Fig. 4A, 50 ng/ml of recombinant TvMIF (rTvMIF) increased parasite survival 1.2-fold at 4 h and 1.3-fold at 8 h after serum starvation. Human MIF has been reported to be secreted during parasitic infection (35, 36). Thus, we tested whether recombinant huMIF (rhuMIF) can also increase parasite survival. We found that 50 ng/ml of rhuMIF induced parasite survival 1.2-fold at 8 h (Fig. 4B). These results support the idea of ability of the parasite to respond to both TvMIF and huMIF, increasing parasite survival, and indicate that T. vaginalis can hijack huMIF to enhance its survival.

### Parasites overexpressing TvMIF can enhance survival of neighboring parasites in co-cultures.

**FIG 5:**
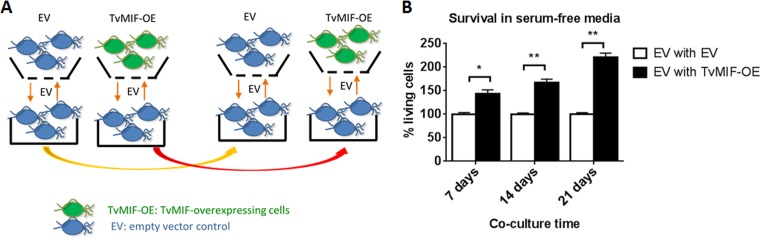
Increased secretion of TvMIF by TvMIF-OE parasites increases survival of EV parasites after co-culture with TvMIF-OE parasites. (A) Scheme of co-culture transwell assay. EV parasites in the wells indicated at the bottom were separated from EV or TvMIF-OE in the wells indicated at the top by a membrane with 0.4-µm pores. (B) Co-culturing EV parasites with TvMIF-OE parasites confers a survival advantage. EV parasites (bottom well in Fig. 5A) were co-cultured with TvMIF-OE parasites (black bars) or EV parasites (white bars) for the number of days indicated on the *x* axis. The EV parasites in the bottom well were then transferred to serum-free media for 24 h, and survival was measured. Error bars represent means ± standard errors. *, *P* value ≤ 0.05; **, *P* value ≤ 0.01. The data represent results from 3 independent experiments, each done in triplicate.

Having established that secreted TvMIF is involved in signaling to parasites, we next tested whether this occurs in an autocrine manner only or can operate in *trans*. To test if secreted TvMIF can enhance the survival of neighboring parasites, we set up a co-culture of EV and TvMIF-OE parasites in a transwell apparatus using complete Diamond’s media. The bottom wells contained EV, and the top wells, separated from the bottom wells by membranes with 0.4-µm pores, contained either EV or TvMIF-OE. Parasites in both the top and bottom wells were passaged into new media daily for 7, 14, and 21 days (Fig. 5A) to maximize exposure of EV to abundant TvMIF secreted from TvMIF-OE. Then, the cultures were switched to serum-free media to induce nutrient stress and we measured the survival of the EV parasites in the bottom wells under conditions of co-culture with either EV or TvMIF-OE parasites. We found that EV parasites co-cultured with TvMIF-OE parasites for 7 days had a 1.4-fold-higher survival rate than those co-cultured with EV control parasites, that those co-cultured for 14 days had a 1.7-fold-higher survival rate, and that those co-cultured for 21 days had a 2.3-fold-higher survival rate and that the survival advantage became more dramatic with increasing numbers of days of co-culturing (Fig. 5B). These data indicate that the abundance of TvMIF secretion from TvMIF-OE parasites enhanced the survival of neighboring EV parasites, possibly exerting this effect via a positive-feedback loop.

### TvMIF inhibits parasite apoptosis.

**FIG 6:**
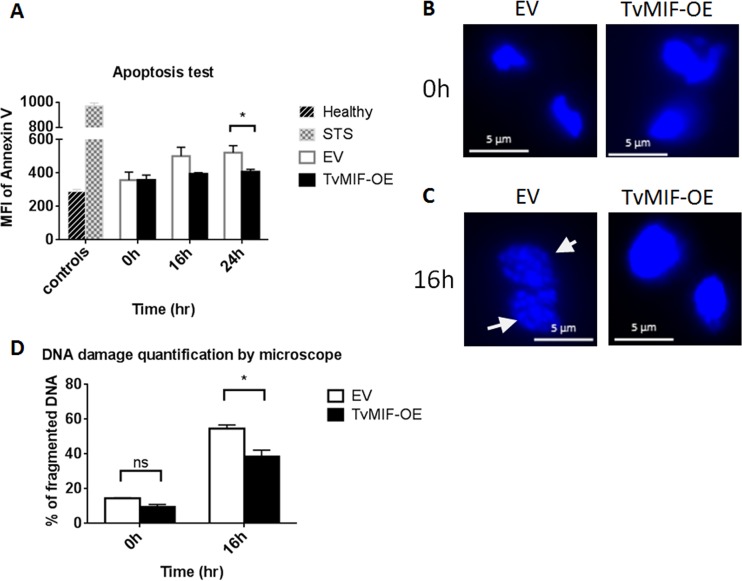
TvMIF inhibits parasite apoptosis. (A) Double staining with annexin V and Zombie Red was used to assess apoptosis. TvMIF-OE parasites (black bars) underwent less apoptosis at 16 h and 24 h after serum starvation than EV parasites (white bars). MFI, mean fluorescence intensity; Healthy, DMSO-treated control; STS, staurosporine (apoptosis inducer positive control). Error bars represent standard errors. *, *P* value ≤ 0.05. (B) At 0 h, both EV and TvMIF-OE nuclei were intact with little damage. (C) Nuclei were stained with DAPI. At 16 h after serum starvation, DNA damage in EV parasites was clearly visible. White arrows indicate examples of DNA fragmentation. (D) DNA damage in EV parasites was significantly greater than that observed in TvMIF-OE parasites after 16 h of serum starvation. The imaging data shown in panels B and C were quantified by counting the number of fragmented DNA in a total of 300 nuclei from both EV and TvMIF-OE parasites. *, *P* value ≤ 0.05. Error bars represent means ± standard errors. All data represent results from 3 independent triplicated experiments.

HuMIF is known to activate anti-apoptotic pathways in human cells (14, 15, 37–39). To test whether TvMIF inhibits the apoptosis of the parasites during serum starvation, we employed a double-staining method similar to the commonly used annexin V and propidium iodide (PI) method (40) except that we replaced PI with Zombie Red as the viability dye to exclude dead cells in the population. Annexin V, which labels externalized phosphatidylserine, is used as an indicator of the number of parasites undergoing apoptosis (40). Using this assay, we observed that EV parasites had slightly more apoptosis than TvMIF-OE parasites at 16 h (*P* value = 0.07). Moreover, after 24 h of serum starvation, EV parasites were significantly more apoptotic than TvMIF-OE parasites (*P* value = 0.01) (Fig. 6A). To further validate the apoptotic phenotypes of the serum-starved parasites, we examined the nuclei of EV and TvMIF-OE parasites grown in serum-free media for 16 h, as the nuclei of T. vaginalis are reported to become condensed and fragmented when treated with apoptosis inducers (24, 25). As shown in Fig. 6B and quantified in Fig. 6D, both EV and TvMIF-OE parasites had low levels of DNA fragmentation at 0 h. In contrast, 16 h after serum starvation, ~55% of EV parasites exhibited apoptotic-like DNA fragmentation, whereas only ~38% of TvMIF-OE parasites displayed this phenotype (Fig. 6B to D). These data demonstrate that TvMIF enhancement of parasite survival is associated with a decrease in apoptosis. It is notable that the observed difference between TvMIF-OE and EV parasites with respect to the anti-apoptotic phenotypes seen at 16 and 24 h is not as dramatic as that observed for the survival phenotype (Fig. 1A), indicating that other mechanisms may contribute to survival during serum starvation.

### TvMIF inhibits parasite apoptosis during serum starvation via ROS suppression.

**FIG 7:**
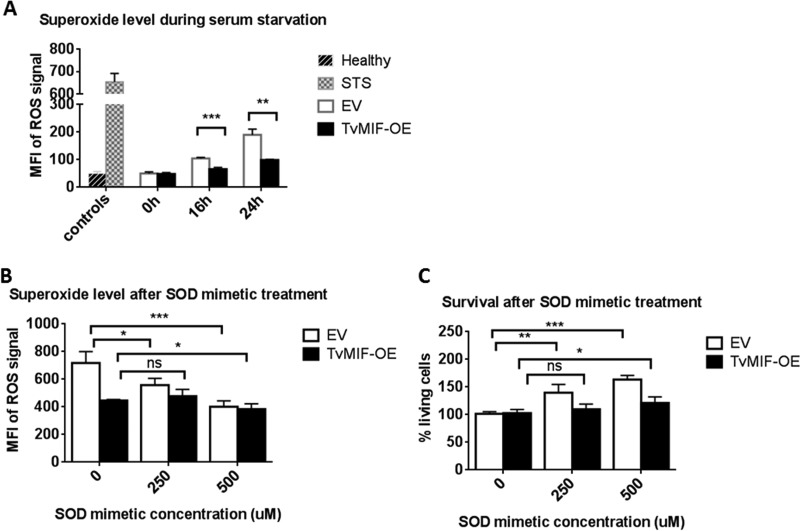
TvMIF inhibits parasite apoptosis under conditions of serum starvation via ROS suppression. (A) The superoxide level detected by using dihydroethidium (DHE) was significantly higher at both 16 h and 24 h in EV parasites than in TvMIF-OE parasites after serum starvation. MFI, mean fluorescence intensity. (B) Superoxide dismutase (SOD) mimetic treatment at time point 0 h decreased the superoxide level in EV at both 250 µM and 500 µM and in TvMIF-OE at 500 µM. (C) SOD mimetic treatment increased EV parasite survival at both 250 µM and 500 µM and TvMIF-OE parasite survival at 500 µM. Ns, non-significant; *, *P* value ≤ 0.05; **, *P* value ≤ 0.01; ***, *P* value ≤ 0.001. All data represent results from 3 independent experiments, performed in triplicate.

Reactive oxygen species (ROS) activate apoptosis signaling in mammalian systems (41), and huMIF is known to inhibit apoptosis by suppressing ROS production (42, 43). To test whether TvMIF-induced survival under conditions of serum starvation is dependent on ROS inhibition, we measured levels of superoxide, a known by-product of ROS signaling, by staining EV and TvMIF-OE parasites with dihydroethidium (DHE). Upon oxidization by superoxide, DHE is converted to fluorescent 2-hydroxyethidium (44) and the intensity can then be used as a measure of ROS levels. We found that TvMIF-OE parasites produced significantly less superoxide than EV parasites when grown without serum for 16 or 24 h (Fig. 7A). To validate the specificity of the DHE signal, we treated EV and TvMIF-OE parasites with the superoxide dismutase (SOD) mimetic manganese(III) tetrakis(1-methyl-4-pyridyl)porphyrin (MnTmPyP) during serum starvation. MnTmPyP significantly decreased the superoxide signal in EV parasites at both 250 µM and 500 µM and in TvMIF-OE parasites at 500 µM (Fig. 7B). The less dramatic effect that MnTmPyP had on TvMIF-OE parasites is consistent with the ability of TvMIF-OE parasites to suppress ROS production prior to the treatment. In addition, the survival of EV parasites was strongly enhanced by MnTmPyP treatment, whereas TvMIF-OE parasite survival was increased only slightly by the addition of 500 µM MnTmPyP (Fig. 7C). The low increase in survival of TvMIF-OE parasites resulted from normalizing the percentages of living cells of treated TvMIF-OE and untreated TvMIF-OE parasites (Fig. 7C) as the untreated TvMIF-OE parasites had higher survival rates than EV. Together, these data indicate that inhibition of superoxide by TvMIF contributes to parasite survival under conditions of serum starvation.

### Gene knockout (KO) of TvMIF severely reduced parasite survival in serum-free media.

**FIG 8:**
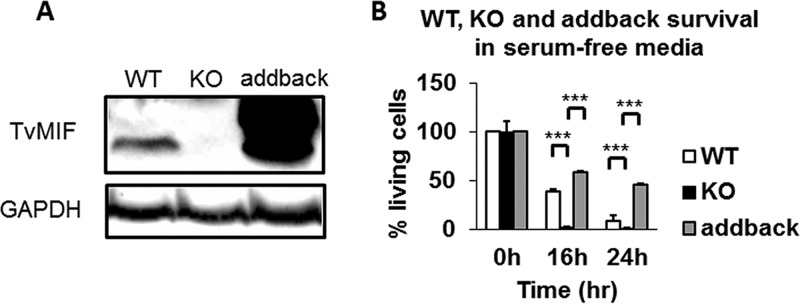
Parasites with the TvMIF gene knocked out have significantly less survival than the wild-type (WT) parasites. (A) Immunoblot using anti-TvMIF antibody to confirm the loss of TvMIF in knockout (KO) parasites and its presence in addback parasites. GAPDH is the loading control. (B) Knockout of TvMIF in the parasite severely reduces the survival of the parasite under conditions of serum starvation. Adding back of TvMIF in knockout parasites that exogenously overexpress the protein restores and enhances the survival phenotype, reminiscent of the increased survival seen with TvMIF-OE. All time points are normalized to time point 0 h for each parasite. Data are means ± standard errors of results from triplicates, and data from 1 of 3 independent experiments are shown. *, *P* value ≤ 0.05; **, *P* value ≤ 0.01; ***, *P* value ≤ 0.001.

After completing the work described above, using TvMIF-OE and EV parasites to study the role of TvMIF in parasite survival under nutrient stress, we succeeded in developing CRISPR-Cas9 methods to knock out (KO) genes in T. vaginalis (4). These methods allowed us to KO TvMIF in the parasite (4). As shown in Fig. 8A, immunoblot analyses using the anti-TvMIF antibody confirmed that TvMIF was depleted in the TvMIF KO parasites. To test whether the effects observed in comparisons of wild-type and KO parasites were specific to the loss of TvMIF, we restored TvMIF to the KO parasites (referred to here as "addback parasites") to test whether this rescued the KO phenotype and caused reversion to that of the wild-type parasites. Immunoblot analysis of addback parasites where TvMIF was overexpressed on a plasmid showed that this resulted in greater expression of TvMIF than was observed in the wild-type parasites (Fig. 8A). Testing the wild-type, TvMIF KO, and addback parasites for survival under conditions of serum starvation, we found that TvMIF KO parasites had 16-fold less and 9-fold less survival than the wild-type parasites and 23.7-fold and 27.7-fold less survival than the addback parasites after 16 h and 24 h under conditions of serum starvation, respectively (Fig. 8B). The increase in survival of addback parasites relative to wild-type parasites is consistent with the higher levels of TvMIF expressed in addback parasites, due to the gene’s presence on a multicopy plasmid. To further validate the survival effect of TvMIF, we added rTvMIF to TvMIF KO parasites in serum-free media and found that 100 ng/ml rTvMIF increased the survival 1.7-fold after 24 h, compared to vehicle control (Fig. S4). Together, these results provide definitive evidence that TvMIF plays a crucial role in resistance to death of the parasite under conditions of nutrient stress.

10.1128/mBio.00910-18.4 FIG S4  Recombinant TvMIF increases the survival of the parasite under conditions of serum starvation. rTvMIF (100 ng/ml) or PBS (indicated as 0 ng/ml) was added to the TvMIF KO parasites grown without serum at time point 0 h and then incubated for 24 h. Quadruplets were done under each set of conditions. ***, *P* values < 0.001. Download FIG S4, TIF file, 1.3 MB. Copyright © 2018 Chen et al. 2018 Chen et al. This content is distributed under the terms of the Creative Commons Attribution 4.0 International license.

## DISCUSSION

We have established a role for TvMIF in the survival of the parasite under adverse conditions. Although MIF has been widely studied in mammals, the function of this conserved protein in other eukaryotes is largely unknown. We demonstrated that overexpression of TvMIF increases parasite survival under conditions of nutrient starvation, which may be important for the parasite to maintain chronic infection in the constantly changing vaginal environment (5). Nutrient starvation was also shown to induce the expression and secretion of TvMIF by the parasite. By co-culturing parasites that overexpress TvMIF (TvMIF-OE) with empty vector (EV) parasites that do not, secreted TvMIF was shown to enhance the survival of EV parasites upon prolonged co-culturing, suggesting the presence of an intracellular positive-feedback pathway by which uptake of TvMIF triggers an increase in TvMIF expression and secretion. We were ultimately able to knock out (KO) TvMIF and to show definitively that the loss of TvMIF severely altered the ability of T. vaginalis to survive under conditions of nutrient stress.

We found that TvMIF inhibits parasite apoptosis during serum starvation, consistent with the increased survival phenotype conferred on TvMIF-OE parasites. These data imply that nutrient deprivation during infection induces parasite apoptotic pathways and that increased production of TvMIF may allow the parasite to survive, awaiting more favorable conditions within the urogenital tract. The ability of huMIF to inhibit apoptosis and to contribute to oncogenesis is well established (15, 38, 45–48). This functional conservation between huMIF and TvMIF is remarkable given their evolutionary divergence. Our findings are consistent with host-parasite interactions providing an environment that allowed co-evolution of the two proteins.

Previous studies indicated that T. vaginalis undergoes apoptosis when treated with drugs known to induce apoptosis in mammalian cells. Treatment with staurosporine (STS) led to DNA condensation and staining with annexin V, both indicators of apoptosis (24). The survival of T. vaginalis under conditions of iron depletion, glucose restriction, or serum starvation has also been described, followed by examination of the resulting differential gene expression (49–51). Re-evaluation of these data revealed that under conditions of glucose restriction, TvMIF mRNA is upregulated by 3.8-fold at 12 h and 1.3-fold at 24 h, indicating that TvMIF may be involved in enhancing parasite survival under conditions of glucose starvation (50). In contrast, RNA analyses of parasites grown under conditions of iron depletion revealed that TvMIF mRNA levels were 2.5 times lower under iron-deficient conditions than under iron-rich conditions (49). However, the time point used to extract RNA for the transcriptome sequencing (RNA-seq) analysis was not stated. Therefore, it is possible that TvMIF was upregulated to exert its survival effect at a different time during the analyses.

Although the studies on RNA regulation under glucose-deficient and iron-deficient conditions (49, 50) provide insights into potential pathways that induce parasite survival, prior to this study, no survival factor had been characterized or manipulated by genetic approaches to directly address mechanisms underlying the survival phenotype. Our study results show that the induction of parasite survival by TvMIF via the inhibition of apoptosis is accompanied by reactive oxygen species (ROS) suppression. These data confirm the evolutionary conservation of apoptosis inhibition pathways, as ROS has been shown to activate apoptosis signaling in mammalian systems (41) and huMIF is known to inhibit apoptosis by suppressing ROS production (42, 43).

Induction of apoptosis in the parasitic protists *Leishmania* spp., Trypanosoma cruzi, and Trypanosoma brucei is accompanied by an increase in ROS (52–57). However, whether the modulation of apoptosis by MIF has been generally conserved between protists and mammals, as appears to be the case for ROS, is yet to be determined. In this regard, it is notable that the known roles of MIF in Xenopus laevis and Caenorhabditis elegans are not directly related to survival under conditions of stress (58, 59). The main functions of other parasite MIFs have been shown to be focused on their effects on hosts. Leishmania major MIF (LmMIF), *Plasmodium* species MIF, Toxoplasma gondii MIF, and Entamoeba histolytica MIF can bind huMIF receptor CD74 and modulate host immune responses, activate huMIF pathways such as the extracellular signal-regulated kinase/mitogen-activated protein kinase (ERK/MAPK) pathways, and affect the infection outcome (10, 18, 20, 60, 61). LmMIF and Plasmodium yoelii MIF also inhibit apoptosis of host macrophages by activating mammalian survival pathways (10, 61, 62). However, the possible anti-apoptotic effect of parasite MIFs on these parasites is yet to be examined.

We found that both TvMIF and huMIF confer cell survival, which may be the result of host-parasite co-evolution. This notion is supported by our previous studies showing that TvMIF and huMIF induce the same signaling pathways in human cells, conferring an anti-apoptotic and increased-growth phenotype (17), as well as by the data reported here indicating that addition of rhuMIF induces parasite survival under conditions of serum starvation. The latter observation implies that secreted huMIF could also directly affect parasite survival under adverse conditions during infection.

TvMIF was originally identified in the T. vaginalis exosomal proteome (31). HuMIF is also present in the exosomal proteome derived from a variety of human cell types (63–71). The effect of huMIF on cell survival has been shown to be mostly focused on soluble huMIF (14, 72, 73). However, Costa-Silva et al. showed that pancreatic ductal adenocarcinoma-derived exosomes with abundant huMIF play a role in inducing liver metastasis, although they did not test whether exosomal huMIF directly triggers anti-apoptotic pathways (71). Further analyses will be required to determine whether exosomal huMIF or TvMIF is capable of triggering anti-apoptotic pathways when exosomes fuse and deliver their protein cargo into human and/or trichomonad cells. Likewise, future transcriptomic and/or proteomic analyses should assist in identifying the anti-apoptotic pathway(s) triggered by TvMIF.

## MATERIALS AND METHODS

### T. vaginalis cell culture and transfection.

T. vaginalis strain B7RC2 (ATCC 50167) was cultured in Diamond’s medium supplemented with 100 U/ml penicillin and 100 µg/ml streptomycin (Thermo Fisher Scientific), 180 µM ferrous ammonium sulfate (Fisher), 28 µM sulfosalicylic acid (Fisher), and 10% horse serum (Sigma) (complete Diamond’s media) (26). TVAG_219770 (TvMIF) was overexpressed in Master-Neo-(HA [hemagglutinin])_2_ plasmid and transfected as previously described (17). The plasmid was maintained with 100 µg/ml of G418 (Gibco) for selection. Parasites were cultured at 37°C and passaged daily for 2 weeks or less.

### Serum starvation assay.

T. vaginalis was cultured overnight in complete Diamond’s media (26). The parasites were then pelleted by centrifugation and resuspended at 10^6^ cells/ml in the same media, with the exception that no horse serum was added (serum-free Diamond’s media), and incubated at 37°C at the time points indicated. At each time point, 500 µl of culture was taken from each sample and read on a FACS instrument. The survival rates of the parasites were determined by excluding dead cells using Zombie Red viability dye at a 1:1,000 dilution in PBS (BioLegend) and quantitated using CountBright Absolute Counting Beads (Thermo Fisher Scientific) with a BD LSRFortessa cell analyzer (see Fig. S2 in the supplemental material). The percentages of living cells at all of the time points were normalized to the level at time point 0 h, which was set as 100%.

### Density stress assay.

EV and TvMIF-OE parasites were grown to mid-log phase (10^6^ cells/ml) overnight and concentrated to 10^7^ cells/ml or 2 × 10^7^ cells/ml in complete Diamond’s media. These parasites were then incubated for 4 h or 8 h. The cells were subjected to FACS analyses using a BD LSRFortessa cell analyzer. The percentages of dead cells were determined by gating the Zombie Red-positive population and determining its percentage relative to the total cell population.

### Growth assay.

EV and TvMIF-OE parasites (4 × 10^4^ cells/ml) were cultured in complete Diamond’s media. At 12 h and 24 h, 500 µl of each sample was taken for FACS analyses to determine the numbers of living cells. Numbers of living cells were determined as described for the serum starvation assay.

### Secretion assay.

The parasites were grown to mid-log phase overnight in complete Diamond’s media, collected, and resuspended in PBS–5% sucrose at 10^6^ cells/ml. The parasites (5 × 10^8^) were incubated at either 16°C or 37°C for 2 h. The 16°C condition was used for the secretion inhibition control. After the incubation, the parasites were spun at 3,200 rpm for 10 min and the cell pellets were collected and lysed in lysis buffer containing 50 mM Tris-HCl (pH 7.5), 2% SDS, and 1× Halt protease inhibitor cocktail (Thermo Fisher Scientific) to make whole-cell lysates (Wcl). The supernatant was passed through a PES Vivaflow crossflow cassette (Sartorious) (MWCO, 100 kDa) to separate the exosomes (Exo) from the non-exosomal soluble fraction (NESF). The NESF was concentrated by using Amicon Ultra Centrifugal filters (EMD Millipore) (MWCO, 10 K). The Exo were pelleted at 100,000 × *g* for 70 min. Anti-neomycin phosphotransferase (Neo) (Jackson Laboratory) (1:2,500) and anti-TvMIF antibodies (polyclonal rabbit antisera raised against TvMIF) (1:500) (4) were used as the primary antibodies and anti-rabbit antibody (Jackson Laboratory) (1:25,000) was used as the secondary antibody for both Neo and TvMIF probing.

### Intracellular and secreted TvMIF quantitation.

Anti-TvMIF polyclonal antibody, anti-glyceraldehyde 3-phosphate dehydrogenase (anti-GAPDH) antibody (Cocalico Biologicals) (1:10,000), and anti-Neo antibody (Jackson Laboratory) (1:2,500) were used as the primary antibodies, and anti-rabbit (Jackson Laboratory) was used as the secondary antibody. For intracellular TvMIF quantitation, mid-log-phase parasites were spun down and washed with PBS–5% sucrose–1× Halt protease inhibitor cocktail (Thermo Fisher Scientific). The cells were then lysed in 50 mM Tris-HCl (pH 7.5)–2% SDS–1× Halt protease inhibitor cocktail lysis buffer. Equal total proteins were loaded from whole-cell lysates from 0 h, 16 h, and 24 h. For secreted TvMIF quantitation, T. vaginalis parasites cultured overnight in complete Diamond’s media were collected and resuspended in serum-free or complete Diamond’s media for 3 h or 6 h of incubation. After the incubation, the parasites were resuspended in PBS–5% sucrose for 2 h of incubation for secretion collection. The cells were spun down and collected using the method described for intracellular TvMIF quantitation as the whole-cell lysate (Wcl) control. The supernatant was then concentrated with Amicon Ultra Centrifugal Filters (MWCO = 10 K). Equal total protein amounts were loaded for the Wcl control and secreted fractions for all samples.

### Production and purification of rTvMIF and rhuMIF.

TvMIF and huMIF was cloned into the pET SUMO expression vector with an N-terminal SUMO domain with 6×His tag separately. Both constructs were transformed into BL21(DE3) E. coli (Thermo Fisher Scientific) for protein expression (74). rTvMIF and rhuMIF were purified using a HisPur nickel-nitrilotriacetic acid (Ni-NTA) Spin column (Thermo Fisher Scientific) and dialyzed into 20 mM Tris-HCl (pH 8.0)–150 mM NaCl–1 mM dithiothreitol (DTT). His-tagged SUMO protease (mclab) in combination with SUMO protease buffer (50 mM Tris (pH 8.0), 1 mM DTT) was used to cleave off N-terminal SUMO and the 6×His tag to produce rTvMIF and rhuMIF with their native sequences. SUMO protease and the SUMO domain with the His tag were removed by the use of a HisPur Ni-NTA Spin column. rTvMIF and rhuMIF were then purified by the use of Econo-Pac 10DG desalting prepacked gravity flow columns (Bio-Rad).

### Exogenous rTvMIF and rhuMIF addition and survival assay.

A 50 ng/ml volume of rTvMIF or rhuMIF or PBS vehicle control was added to TvMIF-OE grown in serum-free Diamond’s media. The survival rates were determined 4 h and 8 h after starvation. rTvMIF (100 ng/ml) or PBS vehicle was added to TvMIF KO parasites in serum-free Diamond’s media. The survival rates were determined 24 h after starvation.

### Co-culture in transwell.

Transwell (Corning) with a 0.4-µm-pore-size polycarbonate membrane insert was used. EV or TvMIF-OE (1.8 × 10^5^) was plated in 300 µl of complete Diamond’s media in each individual top insert, and EV (2 × 10^5^) was plated in 1 ml of media in each individual bottom well. The top and bottom cells were passaged and rediluted daily to the concentrations described above in complete Diamond’s media. Every 7 days, the cells were resuspended in serum-free Diamond’s media at the concentration described above and incubated for 24 h for survival tests. The survival rates were determined as described for the serum starvation assays.

### Apoptosis assays.

Zombie Red (1:1,000) and 4.5 µg/ml of fluorescein isothiocyanate (FITC)-annexin V (BioLegend) in annexin V binding buffer (BioLegend) were used to stain parasites grown without serum at 0 h, 16 h, and 24 h, and the cells were subjected to FACS analysis using a BD LSRFortessa cell analyzer. The live parasites were gated by excluding positive Zombie Red signal. Apoptotic levels were determined by analysis of the mean fluorescence intensity (MFI) of annexin V of live parasites. Parasites treated with dimethyl sulfoxide (DMSO) were used as the healthy control, and parasites treated with 4 µM staurosporine (STS) for 16 h composed the apoptotic control.

DNA fragmentation was done by incubating EV and TvMIF-OE in serum-free Diamond’s media and fixing parasites in 4% formaldehyde–PBS. Parasite nuclei were stained with ProLong Gold antifade mountant with 4′,6-diamidino-2-pheylindole (DAPI) (Thermo Fisher Scientific). The slides were then imaged using an Axioskop 2 epifluorescence microscope (Zeiss). The percentage of fragmented DNA was determined by the number of fragmented DNA in a total of 300 nuclei counted in each sample by using ZEN lite software.

### Reactive oxygen species (ROS) detection and superoxide dismutase (SOD) mimetic treatment.

EV and TvMIF-OE parasites were grown in serum-free Diamond’s media as previously described. At 0 h, 16 h, and 24 h, cells were stained with 2 µM dihydroethidium (DHE) (Thermo Fisher Scientific) mixed with PBS at 37°C for 30 min in the dark. The MFI of ROS signal was determined by the MFI of phycoerythrin-Texas Red (PE-Texas Red)-treated live cells gated by the use of a flow cytometer.

For SOD mimetic treatment, a 250 µM or 500 µM concentration of manganese(III) tetrakis(1-methyl-4-pyridyl)porphyrin (MnTmPyP) (Sigma) or DMSO was used to treat parasites grown in serum-free Diamond’s media at time point 0 h. At 16 h, parasites were stained with DHE and CountBright absolute counting beads (Thermo Fisher Scientific) were added to determine the number of live cells. The ROS signal and the percentage of living cells were measured by the use of a flow cytometer.

### Gene knockout and adding back of TvMIF in T. vaginalis B7RC2.

The constructs and reagents used to create the TvMIF knockout were as described by Janssen et al. (4). Adding back of TvMIF to TvMIF KO cells was done by transfection of TVAG_219770 (TvMIF) in Master-Neo-(HA)_2_ plasmid with selection of G418 at the concentration of 900 µg/ml in order to select for the plasmid in the presence of a neomycin phosphotransferase (Neo) gene knock-in at the TvMIF gene locus, replacing the TvMIF gene.
