# Supplementary figures and images for: Trichomonas vaginalis Macrophage Migration Inhibitory Factor Mediates Parasite Survival during Nutrient Stress
Source: mBio. 2018 Jun 26;9(3):e00910-18. doi: 10.1128/mBio.00910-18 (PMC6020296; doi:10.1128/mBio.00910-18)

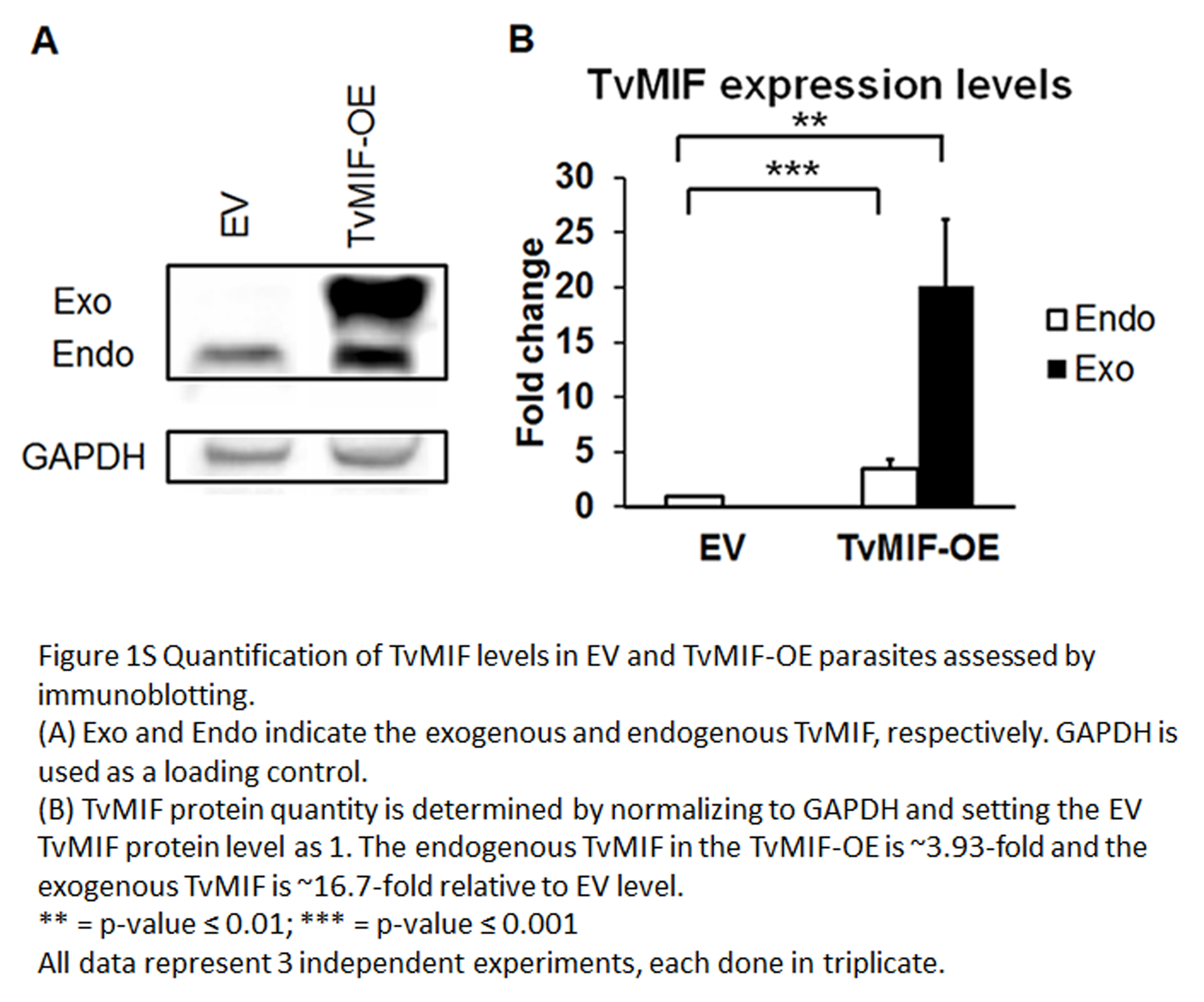

Supplement: FIG S1 [file mbo003183953sf1.tif]

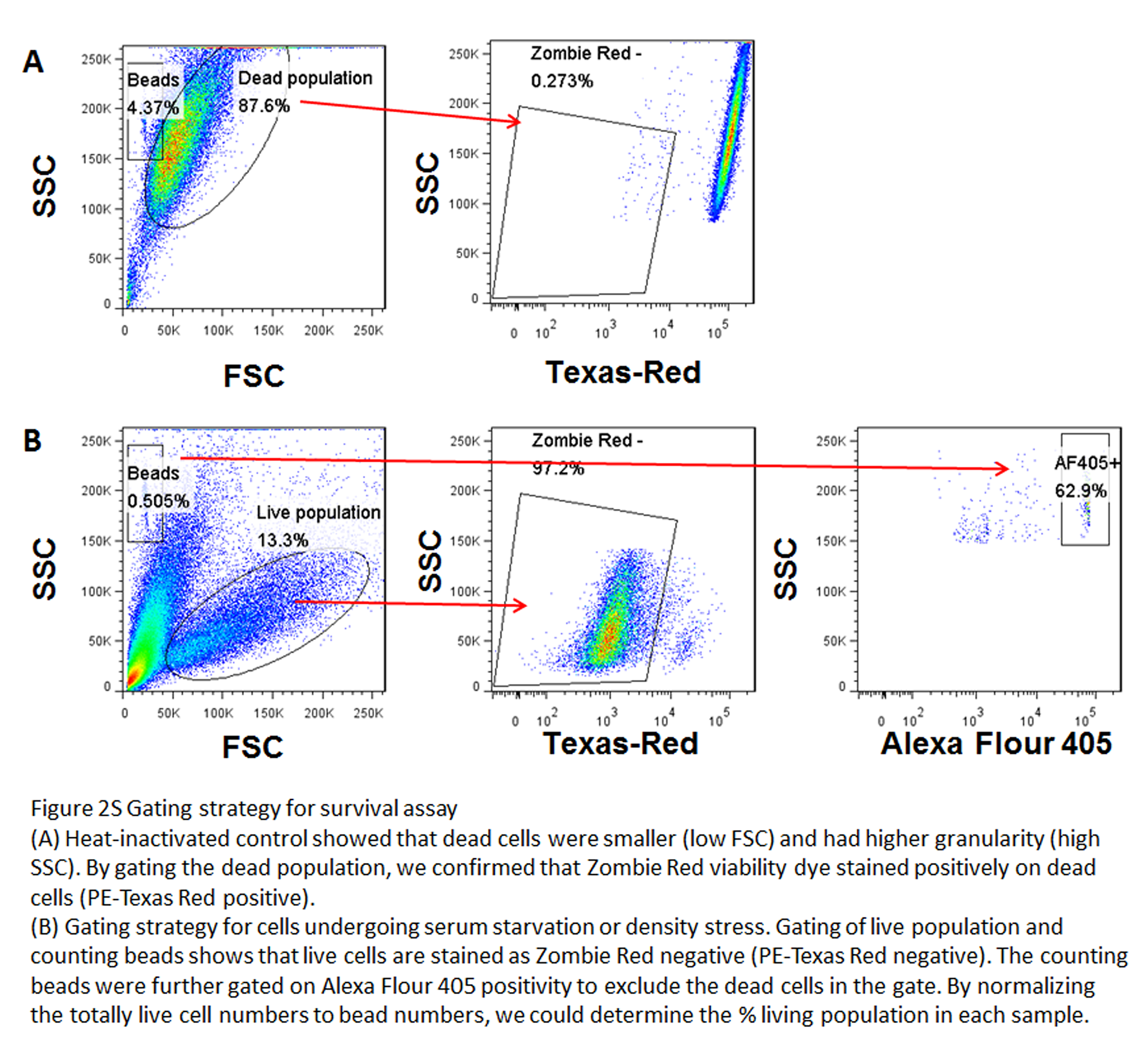

Supplement: FIG S2 [file mbo003183953sf2.tif]

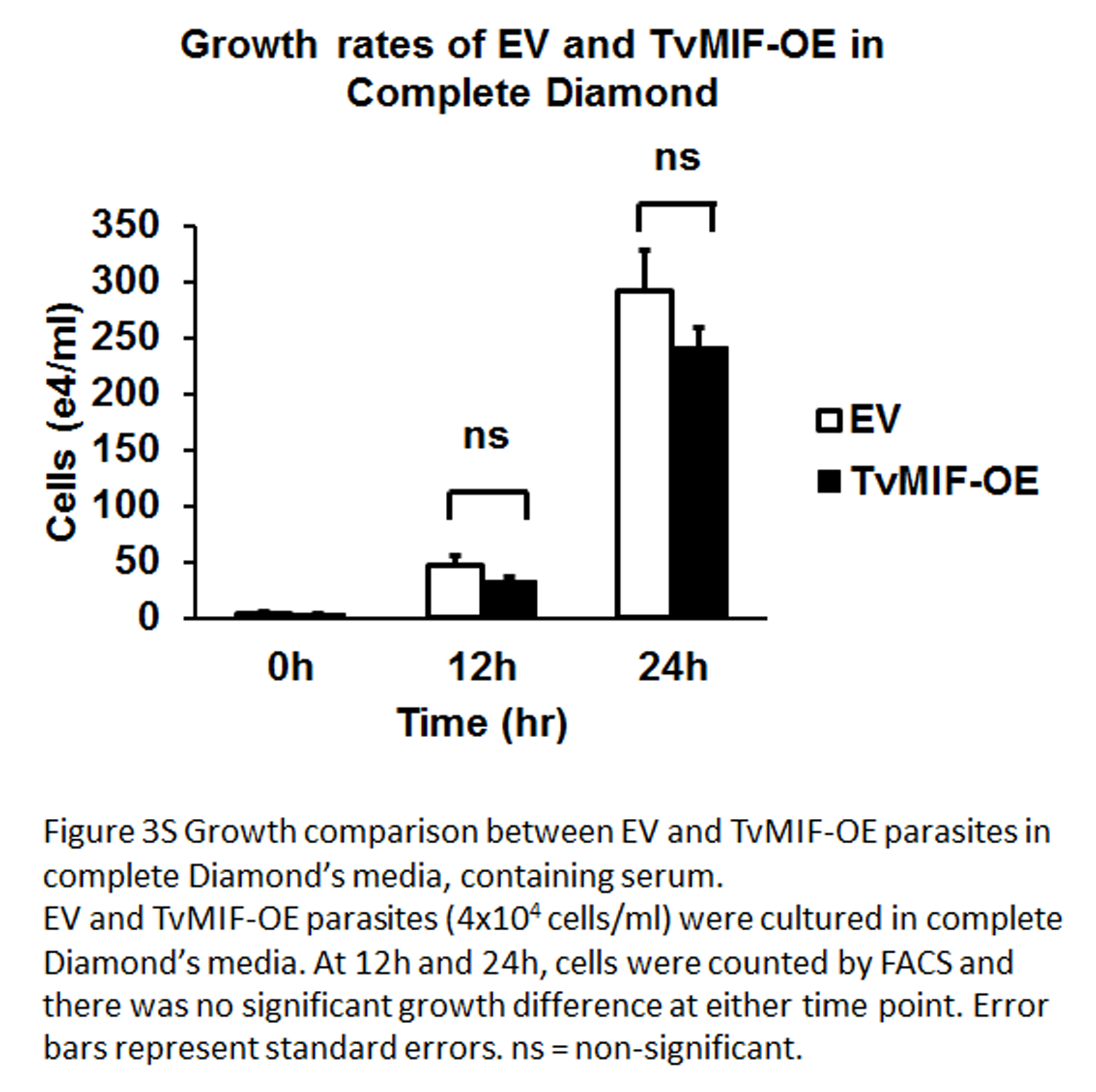

Supplement: FIG S3 [file mbo003183953sf3.tif]

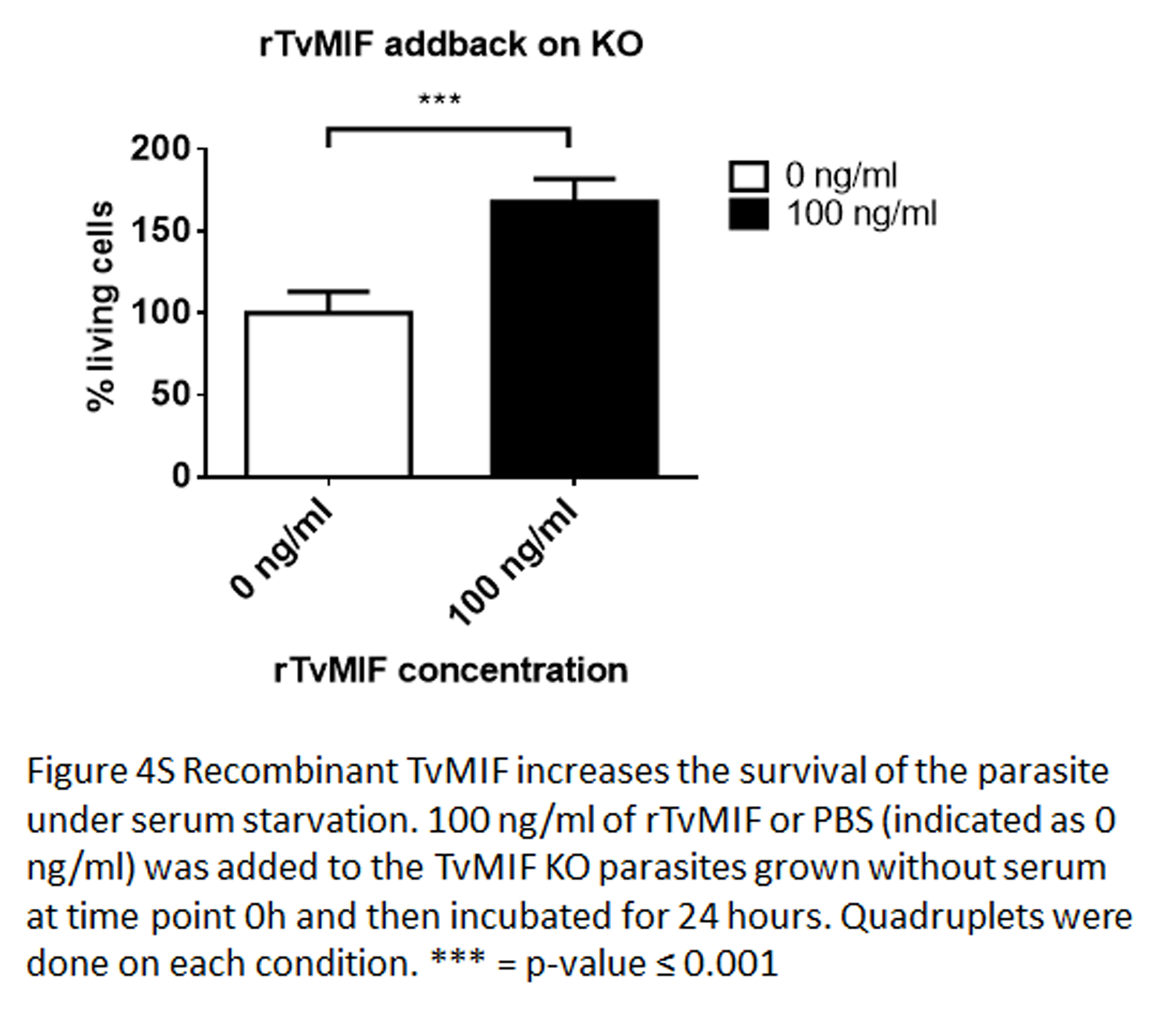

Supplement: FIG S4 [file mbo003183953sf4.tif]
